# Catheter Ablation of Ventricular Arrhythmias Originating From the Pulmonary Sinus Cusp in Pediatric Patients: A Single-Center Retrospective Study

**DOI:** 10.3389/fped.2019.00280

**Published:** 2019-07-09

**Authors:** Tian Liu, Dongpo Liang, Zili Liao, Zhiwei Zhang, Shushui Wang, Shaoying Zeng

**Affiliations:** ^1^Guangdong Provincial Key Laboratory of South China Structural Heart Disease, Department of Pediatric Cardiology, Guangdong Cardiovascular Institute, Guangdong Provincial People's Hospital, Guangdong Academy of Medical Sciences, Guangzhou, China; ^2^Guangdong Provincial Key Laboratory of South China Structural Heart Disease, Department of Cardiology, Guangdong Cardiovascular Institute, Guangdong General Hospital, Guangdong Academy of Medical Sciences, Guangzhou, China

**Keywords:** ventricular arrhythmia, radiofrequency catheter ablation, pulmonary sinus cusp, pediatric patient, ventricular tachycardia, premature ventricular contractions

## Abstract

**Objective:** There are few reports of ventricular arrhythmias (VAs) originating from the pulmonary sinus cusp (PSC) in pediatric patients. Thus, we investigated the ablation of PSC-VAs in pediatric patients.

**Study Design:** Clinical, echocardiographic, and ablation data were reviewed in 10 consecutive symptomatic children who underwent successful ablation of VAs of PSC origin at our center between March 2014 and June 2018.

**Results:** The 10 patients' weights ranged from 29 to 63.5 kg, and all had structurally normal hearts and VAs with left bundle branch block (LBBB) morphologies and inferior axes. The initial ablation was performed in the right ventricular outflow tract (RVOT) or the aortic sinus cusp, which failed to terminate the VAs in nine patients. The successful ablation site was in the right cusp (RC) in seven patients, the anterior cusp in two patients, and the left cusp (LC) in one patient. The earliest potential recorded at the PSC ablation site preceded the onset of the QRS complex during VAs by 29.4 ± 4.9 ms.

**Conclusions:** VAs with a LBBB morphologies and inferior axes may originate within the PSC of children. Ablation was effective and safe for the eradication of VAs originating from the PSCs in children. Due to the particularity of ablations in pediatric patients, mapping of PSCs should be considered when ablation fails in the RVOT.

## Introduction

Ventricular arrhythmias (VAs) are prevalent in pediatric patients and most occur without underlying heart disease. Idiopathic ventricular tachycardia (VT) or premature ventricular contractions (PVCs) with left bundle branch block (LBBB) morphologies and inferior axes in pediatric patients, most commonly originate from the right ventricular outflow tract (RVOT) (1). Radiofrequency catheter ablation (RFCA) has been increasingly used in the treatment of such arrhythmias; however, their high recurrence rate adversely impacts prognosis. Ablation of VAs from the pulmonary sinus cusp (PSC) has recently been reported in adult patients at our center (2), but not in pediatric populations. In this study, we investigated the catheter ablation of outflow tract ventricular arrhythmias, as well as focusing on the ECG characteristics, mapping, and ablation of VAs arising from the PSC in pediatric patients.

## Patients and Methods

### Study Population

Between March 2014 and June 2018, 43 consecutive symptomatic children (28 males, mean age 10.1 ± 2.9 years, mean weight 37.3 ± 13.6 years) with frequent VAs were referred to our department for catheter ablation. All patients had structurally normal hearts. The ECG recorded during the VAs showed LBBB morphologies and inferior axes in all patients. All children had rhythm-correlated symptoms due to frequent PVCs or VTs. Successful ablations were achieved in 40 patients, including 29 in RVOT (72.5%), one in the right aortic sinus cusp (2.5%), and 10 in the PSC (25.0%). Among the remaining three patients, ablation was unsuccessful in two, while ablation could not be performed in the third due to the pain caused by the procedure.

Prior to electrophysiological examination, all patients underwent non-invasive cardiac evaluations, including reviews of family histories, physical examinations, 12-lead electrocardiograms (ECG), 24-h Holter ECGs, and transthoracic echocardiography. All patients had a normal ECG during sinus rhythm, and no structural abnormalities were apparent upon physical examination. The indications for catheter ablation included symptomatic VAs and ineffective treatments with antiarrhythmic drugs. The exclusion of VAs was due to transient reversible causes, such as acute myocarditis or drug toxicity. The cardiac dimensions of children were in accordance with their ages, heights, body weights, and body surface areas. Cardiac dilatation was defined as an echocardiography-measured cardiac cavity size beyond one standard deviation. The hospital ethics committee approved the study, and all patients' guardians provided written informed consent before procedures were performed.

### Electrophysiological Analyses

All antiarrhythmic drugs were withdrawn for a duration of at least five half-lives. Catheters were inserted through the right femoral vein into the right atrium, right ventricle, and PA using fluoroscopic guidance. If the clinical arrhythmia failed to occur spontaneously and was not provoked by the administration of intravenous isoproterenol infusion, programmed ventricular stimulation, and incremental burst pacing were performed at two basic drive cycle lengths with <2 extra stimuli to a minimum coupling interval of 230 ms. During the procedure, intravenous heparin was delivered as a 100 IU/kg bolus dose and again every hour thereafter to maintain an activated clotting time between 250 and 300 s.

### Mapping and RFCA

After the baseline study, a steerable 7.5-F catheter with a 3.5-mm irrigated-tip electrode with 2-5-2 mm inter-electrode spacing (NaviStar ThermoCool, Biosense Webster, Diamond Bar, California), was used for 3-dimensional electroanatomical mapping and ablation. The catheter was advanced, via the 8.5-F SL1 long sheath and introduced through the right femoral vein into the right ventricle. Point-by-point mapping was used to create anatomic maps, and activation mapping was performed during spontaneous/induced VT or PVCs to identify the origin of the VAs. Pace mapping was also used during the electrophysiological study to capture the ventricular myocardium at the site of the earliest activation. The pacing morphology was compared with the spontaneous arrhythmia. A good pace map was defined as a paced QRS morphology that matched the clinical VAs in ≥10 of 12 leads. The suitable ablation target site was defined as the site where the best pace mapping and/or the earliest activation time could be obtained during the VT or PVCs.

In 10 patients, the successful ablation sites were in the PSC. According to QRS morphologies during VAs, initial mapping was performed in the RVOT in nine patients, and in the aortic sinus cusp in one patient. When ablation failed, or suitable ablation sites were not found in the RVOT or aortic sinus cusp, mapping within the PSC was performed. Detailed mapping within the PSC was performed using a reversed U curve of the ablation catheter. The SL1 long sheath was used to advance into the right ventricle to permit a reversed U curve in the PA, which could enhance stability of the catheter. Furthermore, the long sheath helped clockwise or counterclockwise catheter rotation to access the three individual PSCs (2).

The irrigated radiofrequency current was delivered in power-controlled mode, with a preselected temperature of 40–43°C, a radiofrequency energy of 25–30 W, and a flow rate of 17–20 ml/min. If arrhythmias were eliminated, or decreased in frequency by radiofrequency within the initial 15 s, the radiofrequency energy applications were maintained for 60–90 s. Following elimination of the VAs by radiofrequency delivery, the catheter position was evaluated and confirmed by repeat pulmonary arteriograms, right ventriculography, or coronary angiography. However, intracardiac echocardiography was not used to evaluate catheter position. After successful ablation, intravenous isoproterenol infusion was administered, and programmed stimulation was performed to confirm the elimination of clinical arrhythmias. Transthoracic echocardiography was performed to assess pericardial effusion during the procedure.

Successful RFCAs were determined by three criteria: (1) the absence of spontaneous or induced clinical VAs upon completion of the procedure; (2) the absence of VTs or PVCs during 24-h ECG monitoring with the patient not administered antiarrhythmic drugs; and (3) no recurrence of VTs or PVCs in the absence of antiarrhythmic drugs for at least 1 year following the procedure. Transthoracic echocardiography was performed before hospital discharge for all pediatric patients.

### Follow-up

Following ablation, all children received 3–5 mg/kg per day of aspirin for 3 months, but no antiarrhythmic drugs. The follow-up consisted of examinations at an outpatient clinic. Patients were assessed at 1, 3, and 6 months after the procedure, and yearly thereafter. A 12-lead ECG was performed at each visit, while transthoracic echocardiography and 24-h Holter ECG were performed at the 3, 6-month, and annual follow-up visits.

### Statistical Analysis

Data were presented using counts (%) for categorical variables and means ± standard deviations (SD) for normally-distributed continuous variables. The parameters in different groups were compared using the Student's unpaired *t*-test. A two-tailed *p*-value <0.05 was considered statistically significant. Data analyses were performed using SPSS 19.0 (SPSS Inc., USA).

## Results

The characteristics of the 40 pediatric patients, with successful ablations, are given in Table 1. Patient symptoms consisted of dizziness, palpitations, chest distress, or exercise intolerance upon the presentation of VAs. None of the patients experienced syncope at the presentation of VAs. Prior to ablation, all patients received medical therapy, where 30 (75%) were managed with one antiarrhythmic drug, and 10 (25%) were administered more than two consecutive or concomitant antiarrhythmic drugs. The drugs consisted of oral amiodarone, metoprolol, and propafenone. Medical therapy was ineffective in all patients. In 29 patients, the successful ablation sites were in the RVOT (at the posteroseptal region in 12, midseptal region in three, anteroseptal region in eight, the anterior free wall in three, and the posterior free wall in three).

**Table 1:** Characteristics of pediatric patients with successful ablation.

**Characteristic**	**PSC group**	**RVOT group**	**ASC group**
	**(*n* = 10)**	**(*n* = 29)**	**(*n* = 1)**
Age (years)	11.3 ± 2.1	9.4 ± 2.9	12
Male	6 (60)	20 (69.0)	1 (100)
Weight	43.1 ± 10.9	34.3 ± 13.8	32
Age at VAs onset (years)	10.2 ± 1.6	8.2 ± 3.6	10
**CLINICAL VAs**			
Only PVCs	8 (80)	24 (82.8)	1 (100)
PVCs and non-sustained VT	2 (20)	5 (17.2)	0
**MEDICAL THERAPY**			
One single antiarrhythmic drug	6 (60)	23 (79.3)	1 (100)
More than two antiarrhythmic drug	4 (40)	6 (20.7)	0
PVCs burden on 24-h!!! Holter electrocardiogram(%)	32 ± 7.7	32.4 ± 11.4	46.5
**ECHOCARDIOGRAM**			
Normal cardiac cavity size	10 (100)	29 (100)	1 (100)
Left ventricular ejection fraction (%)	71.3 ± 7.5	70.0 ± 6.4	61

*Values are given as no. (%) or means ± SD. PSC, pulmonary sinus cusp; RVOT, right ventricular outflow tract; ASC, aortic sinus cusp; Vas, ventricular arrhythmias; PVCs, premature ventricular contractions; VT, ventricular tachycardia*.

The 10 patients with VAs from the PSC (mean age, 11.3 ± 2.1 years) group included six males and four females, with a mean weight of 43.1 ± 10.9 (range, 29–63.5) kilograms. During electrophysiological analyses, Clinical arrhythmias occurred spontaneously or were provoked by the administration of intravenous isoproterenol infusion in seven patients, while clinical PVCs were induced by programmed ventricular stimulation and burst pacing in three of the 10 patients. A previous ablation had been performed successfully in one patient. The site of termination of the PVC was in the RVOT; however, VAs with the same morphologies recurred during follow-up examinations after the patient's first ablation. A repeat procedure was performed 4 months later.

The initial ablation was performed in the left aortic sinus cusp in one patient, in the RVOT in eight patients, and in the PSC in one patient (Table 2). The initial ablation in the left aortic sinus cusp failed to decrease the VAs in one patient. In the left aortic sinus cusp, the recorded local ventricular activation preceded the QRS onset during VAs by 15 ms, and a good pace map was obtained. In the RVOT, the site was at the posteroseptal region in two patients, at the midseptal region in one patient, at the anteroseptal region in one patient, and at the anterior free wall in four patients. Local ventricular activation recorded at the initial ablation site preceded the QRS onset during the VAs by 20.3 ± 5.2 ms, and a good pace map was obtained at those sites in five patients.

**Table 2:** The initial ablation site and the successful ablation site in 10 patients.

**Patient**	**1**	**2**	**3**	**4**	**5**	**6**	**7**	**8**	**9**	**10**
The initial ablation site	RVOT	RVOT	ASC	RVOT	RVOT	RVOT	RC	RVOT	RVOT	RVOT
The successful ablation sit	RC	AC	AC	RC	RC	RC	RC	LC	RC	RC

*RVOT, right ventricular outflow tract; ASC, aortic sinus cusp; RC, right cusp; AC, anterior cusp; LC, left cusp*.

Ablation in the RVOT resulted in transient suppression in seven patients, with a minor change in the QRS morphology of the PVCs in two patients, and termination in one patient. Mapping within the PSC was subsequently performed in those children.

**Figure 1:**
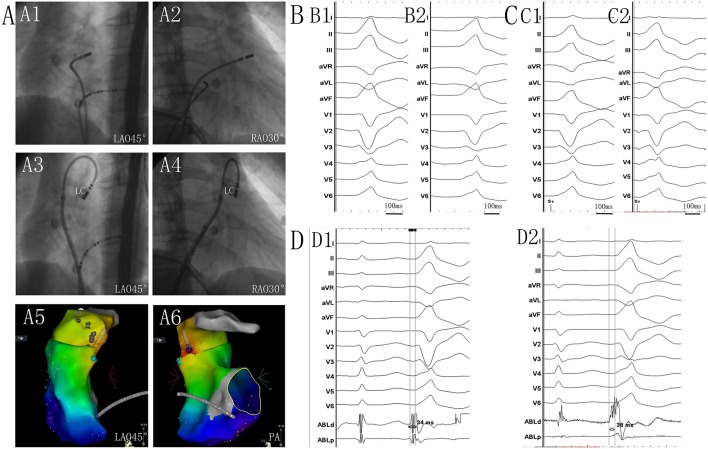
**(A1)** Left anterior oblique (LAO) 45° and **(A2)** right anterior oblique (RAO) 30° views showing the position of the ablation catheter at the site of the anteroseptal region of the right ventricular outflow tract (RVOT). **(A3)** LAO 45° and **(A4)** RAO 30° views showing the position of the successful ablation site within the left cusp (LC). **(A5)** LAO 45° and **(A6)** posteroanterior view showing the anatomic CARTO map of the right ventricle and pulmonary artery (PA). The blue tag represents the earlier activation site in the RVOT anteroseptal region. The red tag represents the earliest site in the LC. **(B1)** Twelve-lead ECG recordings during spontaneous PVC. **(B2)** QRS morphological changes after ablation in the RVOT. Twelve-lead ECG recordings during pacing at the sites of the RVOT anteroseptal region **(C1)**, and at the LC **(C2)**. QRS morphology during pacing in the LC matched that of the spontaneous PVC, and was similar to that of the spontaneous PVC when pacing at the RVOT. **(D1)** The potential preceded the QRS complex by 34 ms during the PVC recording from the catheter at the site of the earlier activation in the RVOT anteroseptal region. **(D2)** The potential preceded the QRS complex by 38 ms during the PVC recording at the site of the earliest activation in the LC. Near- and far-field activations were recorded during sinus rhythm, and its relationship was reversed during PVC.

**Figure 2:**
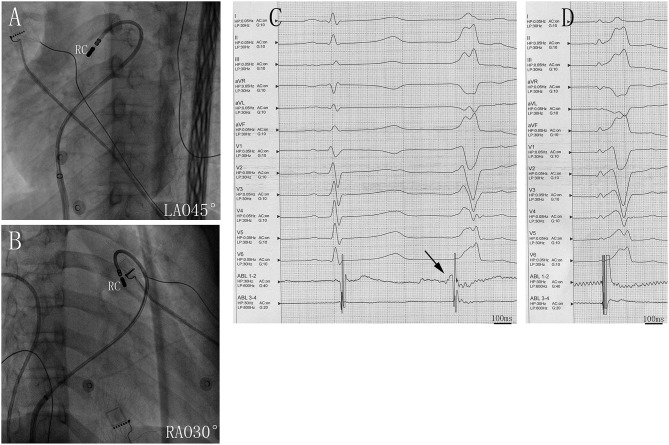
Successful ablation site within the right cusp (RC) in **(A)** left anterior oblique (LAO) 45° and **(B)** right anterior oblique (RAO) 30° views. **(C)** Twelve-lead ECG and ablation catheter (ABL 1-2) recordings during spontaneous PVCs. The potential preceded the QRS complex at the earliest activation site in the RC. **(D)** Twelve-lead ECG recordings during pacing at RC sites with QRS morphologies matching that of spontaneous PVC. Abbreviations are the same as in Figure 1.

**Figure 3:**
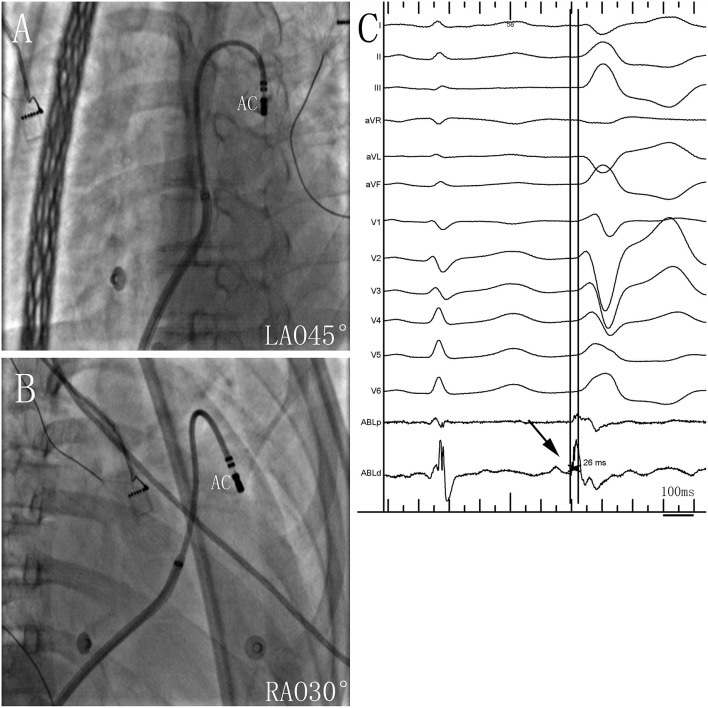
Successful ablation site within the anterior cusp (AC) in **(A)** left anterior oblique (LAO) 45° and **(B)** right anterior oblique (RAO) 30° views. **(C)** Twelve-lead ECG and ablation catheter (ABL d) recordings during spontaneous PVCs. The potential preceded the QRS complex by 26 ms at the earliest activation site in the AC. Abbreviations are the same as in Figure 1.

Two ventricular activation components, including near-field and far-field activation, were recorded at the earliest site in all patients during sinus rhythm, and its relationship was reversed during VAs. When the earliest ventricular activation was identified within the PSC on the bipolar recording, simultaneous activation in the unipolar recording demonstrated the QS morphology. The sharp potential recorded at the PSC ablation site preceded the onset of the QRS complex during VAs by 29.4 ± 4.9 ms, which was significantly greater than the earliest activation time recorded in the RVOT and aortic sinus cusp (*p* <0.05). Pace mapping was performed at the site of the earliest ventricular activation in all patients, with good pace maps obtained in seven patients (64%). VAs originated from the right cusp (RC) in seven patients (70%) (**Figure 2**), the anterior cusp (AC) in two patients (20%) (**Figure 3**), and the left cusp (LC) in one patient (10%) (Figure 1; Table 2). Fluoroscopy was used in all patients and the mean fluoroscopy time was 10.2 ± 6.4 min. The mean procedure duration was 131.3 ± 27.5 min.

The ECG recorded during the VAs revealed a LBBB morphology and inferior axis in all patients (Figures 1–3). The QRS duration was 126.3 ± 17.3 ms in lead V1. Among patients with structurally normal hearts, the precordial transition lead was often observed at V3 and V4, occurring from V3 in three VAs, from V4 in six VAs, and from V5 in one VA. There was a notch on the R-wave in the inferior leads in six patients. A QS-wave was present in avR and avL in all patients.

No complications occurred during ablation. At a mean follow-up of 2.7 years (1–4.5 years), all patients in the study were alive. All patients were free of antiarrhythmic drugs and free of VAs, with no arrhythmia recurrence. Echocardiographic examinations showed left ventricular function was normal in all patients.

## Discussion

The main finding of this 10-patient study was that VAs with LBBB morphologies and inferior axes may have originated within the PSC in children. Ablation was effective and safe for the eradication of VAs originating from the PSC in children. Because of the particularity of ablation in pediatric patients, mapping in PSCs should be considered when ablation fails in the RVOT. In all patients, two ventricular activation components were recorded at the successful ablation site within the PSC.

PVCs are most commonly seen in healthy children. When the PVCs are isolated, they rarely need treatment (3). In studies of adults with structurally normal hearts, frequent VAs can increase the risk of left ventricular dysfunction, and are associated with PVC-induced cardiomyopathy when the burden of PVCs is at least 10% (generally 20–30%) (4, 5). Some studies in children have reached similar conclusions (6, 7). When frequent PVCs have correlated symptoms, or when VAs reduce the left ventricular function, or have hemodynamic compromise, VAs should be treated using drug therapy or ablation (3, 8) in children. All participants in this study had rhythm-correlated symptoms due to VAs, and medical therapy was ineffective.

In adult patients, although VAs can originate from almost any site in the ventricle, they are most commonly located in the RVOT and other sites, including the left ventricular outflow tract, the mitral and tricuspid annuli, the PA, and the aortic sinus cusp. Similar electrophysiological findings have been reported in pediatric patients (9).

Myocardial extensions into the great arteries above the semilunar cusps—or intercuspal—are likely to be secondary to incomplete myocardial regression, and may provide the substrate for PVCs. In previous studies, ventricular myocardial extensions into the PA—beyond the ventriculo-arterial junction—were relatively common, with a prevalence of 17–74% (10, 11). Using direct intracardiac echocardiography visualization, Liu et al. confirmed that 46% of RVOT-VA foci were localized in the PA (median 8.2 mm above PV), indicating that those arrhythmias should be reclassified as pulmonary arterial arrhythmias. Myocardial extensions may provide a substrate for PVCs, since some individuals with myocardial extensions do not experience VAs (12).

Ablation at and around the ventriculo-arterial junction is being increasingly practiced. Some studies demonstrating that the site of origin for outflow tract VAs may be truly intramural, and the ablation strategy chosen is based on proximity and safety (13). Thus, wherever ablation occurs, including in the RVOT, the left ventricular outflow tract, the aorta, or the PSC, it would provide a safe site for ablation. When arrhythmias cannot be eliminated after ablation in the RVOT, subsequent mapping and ablation should be performed in the PA, the aorta, or the PSC. Ablation in PSC would provide a new alternative methodology for the elimination of VAs. Liao et al. reported that in 11% of adults, idiopathic outflow tract-like VAs treated by RFCA could arise from PSCs and be successfully ablated within the PSC (2). Additionally, Zhang et al. reported that ablation within the PSCs effectively eliminated 90% of unselected idiopathic RVOT-type VAs (14). To date, there are no published data on the characteristics and ablation of VAs originating from the PSC in pediatric patients. In this study, we found that in 25% of pediatric patients, idiopathic outflow tract-like Vas, treated by RFCA, were effectively ablated within the PSC.

Activation originating from the PSC is followed by conduction through a pathway to the RVOT. Similar to the interruption of conduction in an accessory atrioventricular pathway, the interruption of conduction either at the beginning or exit of the PSC in the RVOT may abolish VAs (15). Zhang et al. hypothesized that PSC-VAs could be eliminated in the RVOT in some patients when the RVOT exit site was narrow. However, when the exit site is broad or there is an alternative pathway, insufficient ablation may alter the activation impulse and change the QRS morphologies of the PVCs (14, 15). In the present study, the initial ablation was performed in the RVOT in eight patients, resulting in a small change in the QRS morphologies of the PVCs in two patients. Furthermore, the myocardial extensions always had a narrow and thin distal end at the PSC, which may have benefited successful ablations. In this study, consolidating ablation was performed in the PSC after ablation in the RVOT in one patient. All VAs were successfully ablated within the PSC, and in all patients, two ventricular activation components were recorded at the successful ablation site. The sharp potential recorded at the successful ablation site preceded the onset of the QRS by 29.4 ± 4.9 ms, during VAs. The sharp potential was recorded late during sinus rhythm before ablation, and disappeared after ablation. However, most of the local electrograms at the earliest activation sites in the RVOT exhibited a single component. The discrete potential recorded within the PSCs during the VAs and sinus rhythm was characteristic of PSC-VAs. The latter phenomenon was consistent with a report by Liao et al. (2).

The ECG characteristics of the VAs were difficult to distinguish between VAs of RVOT and PSC origins. In our experience, mapping and ablation in the RVOT should be the first consideration in children, as the catheter may injure the valve when approaching the PSC through the pulmonary valve. Mapping in the PSC or left ventricular outflow tract should be considered when ablation fails, or suitable ablation sites are not found in the RVOT. When performing ablation in the RVOT or the PSC, attention must also be paid to the damage caused to the left main coronary artery (16). Mapping and ablation in the PSC was facilitated in our patients by using a reversed U curve ablation catheter that was supported by a long sheath. The weight of the smallest child undergoing ablation within the PSC was 29 kg, and there is limited experience with ablation in the PSCs of smaller children. We suggest that mapping and ablation at the PSC should be performed cautiously in children, and only be performed at experienced and skilled cardiac electrophysiology centers.

The ECG characteristics of VAs originating from PSCs have rarely been reported. In our research of children with structurally normal hearts, the ECG recorded during VAs showed LBBB morphologies and inferior axes with the transitional zone often observed at V3 and V4. Additionally, a notch on the R-wave in the inferior leads was observed in some patients, similar to that reported in adults by Liao et al. (2). These authors also reported smaller R-wave amplitudes in the inferior leads, with longer QRS durations, and larger R-waves in lead I in RC-Vas, compared to that of the AC-VAs and LC-VAs. However, the smaller number of cases in our study precluded comparisons of ECG characteristics between VAs originating from the PSCs in children.

## Limitations

The present study was limited by its single center, retrospective design with small sample size. Those limitations also precluded comparisons of ECG characteristics between PSC-Vas. However, most pediatric studies reported thus far have smaller sample sizes.

In this study of a pediatric population, the vast majority of VAs originating within the PSC exhibited LBBB morphologies, with inferior axes. Ablation was effective and safe for the eradication of VAs originating from the PSC in children. This study established a foundation for ablation in PSCs in children at cardiac electrophysiology centers. Because of the particularity of ablation in pediatric patients, mapping in the PSC should be considered when ablation fails in the RVOT. Additionally, mapping and ablation at the PSC should be performed cautiously in children.

## Data Availability

All datasets generated for this study are included in the manuscript and/or the supplementary files.

## Ethics Statement

This study was carried out in accordance with the recommendations of Guangdong provincial peoples hospital ethics committee with written informed consent from all subjects. All subjects gave written informed consent in accordance with the Declaration of Helsinki. The protocol was approved by the Guangdong provincial peoples hospital ethics committee.

## Author Contributions

SZ has obtained the approval of all other co-authors to submit the manuscript to your journal. All the authors have contribution to the manuscript.

### Conflict of Interest Statement

The authors declare that the research was conducted in the absence of any commercial or financial relationships that could be construed as a potential conflict of interest.
